# Atlanta Academy of Medicine

**Published:** 1873-12

**Authors:** C. A. Simpson

**Affiliations:** Reporter


### Atlanta Academy of Medicine.

C. A. SIMPSON, M. D., REPORTER.

JUNE 16TH, 1873.

Dr. W. S. Armstrong, Vice-President, in the chair.

Dr. Armstrong reported case—called at noon, on Thursday, to see a lady 45 years old, rather fleshy; found her in convulsions; attendants reported her having had some fifteen paroxysms before his arrival; she had a convulsion just after he reached the house.

The previous evening she had eaten some cherries; in the morning she was seized with vomiting and purging, and about ten A.M. the convulsions occurred.

She was bled at once to 24 ounces, and had an active purgative. The blood was dark and coagulated at once. The convulsions ceased as soon as the blood began to flow. Pupils were dilated before the bleeding and contracted afterwards. She soon began to rally and show signs of recognition.

She was seen again in the evening and was quite rational. She had some dysenteric trouble; discharges a little bloody.

He would call attention to the prompt effect of bleeding. Has two cases, now in mind, which he saw with two other physicians who objected to bleeding. Thinks both would have recovered if they had been bled promptly. Never saw a case like these that did not improve after bleeding.

Dr. Baird asked if this attack was due to indigestion, and if it was not rare for convulsions, at this period of life, to be caused by intestinal irritation?

Answer—Thinks it not *very* rare, though perhaps not very frequently encountered. Has seen other cases of convulsions in adults, undoubtedly caused by intestinal irritation.

Dr. J. G. Westmoreland called yesterday to see a lady with the usual symptoms of cholera-morbus; vomiting, purging, feeble pulse, cold sweat—no cramps.

TREATMENT.—Morphia placed on tongue was vomited; then gave 5 grains blue mass with opium, which was retained, and in three or four hours the vomiting and purging ceased.

Dr. Baird asked why he gave the blue pill?

Answer—To allay the vomiting and bring the system under the influence of mercury. Thinks mercury should be given in the initiatory stage of diarrhœa. Believes the disease to be due, in every case, to inaction of the liver.

Dr. Wilson remarked that it was a popular belief, that cholera was confined to lime-stone regions of country, and asked the opinion of the members on this point.

Dr. Rauschenberg referring to the origin and spread of cholera, remarked that it was caused by a vegetable fungus upon the rice plant in India, and through the agency of the trade-winds prevailing in that region the spores of the fungus are diffused through the atmosphere and cause the prevalence of the disease. It is believed in Europe that the disease is not propagated there except by the agency of some cholera patient, and that the infectious particles are present in the discharges. In experiments upon dogs, if the cholera discharges be injected under the skin the symptoms and pathological changes occur as in cholera.

This opinion is entertained by Neimeyer and other prominent members of the profession in Germany. Dr. R. is of the opinion that the only way to prevent the spread of cholera, is by rigid quarantine. The development of the fungus is contingent upon certain extraneous conditions, i.e. heat, moisture, porous soil, vegetable and animal decomposition, etc. Sewerage from privies is one of the most potent agencies. Deposits from rivers and bottom-lands containing a great deal of moisture favor its development. Rock covered by only a few inches of soil and hard soils, in which there is but little moisture, and cities upon a primitive formation, as for instance Atlanta, are not so favorable to its development as those along water courses, as the Mississippi, the Ohio, etc.

It is an established fact, that if the sewerage from privies is disseminated through moist soil, the spores are developed by heat and given off into the air; and you will find the disease following certain water courses, etc. Cholera has been known to break out on board a vessel at sea, where there was no soil or climatic conditions to develop it. If the opinions expressed be correct, the disease must have been brought on the vessel in clothing, merchandise or in some other way.

———

JUNE 23D, 1873.

Dr. W. S. Armstrong, Vice-President, in the chair.

Dr. Baird reported a case of hysteria of unusual duration—Thursday night called to see a negro girl eighteen years old; very much emaciated; menses irregular for five years; last three or four months menstrual function suspended. Efforts were made to arouse her by the application of heated irons to the surface, but with only momentary results. The patient died the following day from asthenia.

Dr. Simpson reported the extraction of a fragment of iron nail, from the eye-ball.

---

JULY 7TH, 1873.

Dr. J. G. Westmoreland, Vice-President, in the chair.

Dr. V. H. Taliaferro reported the following case from his clinic:

Negro woman aged fifty to sixty; number of years since menses ceased; bad health for about a year; somewhat reduced in flesh and strength; no appetite. Complains of pain in the back and profuse, offensive leucorrhœa. Examination: vagina contained much offensive pus. Taxis; uterus large, flabby, soft; cervix small and almost obliterated. Speculum; cervix pale, no congestion, os patulous; could almost introduce the finger into uterus. Introduced forceps and slightly opened the os when a large quantity of pus gushed out, requiring three or four sponges to absorb it. Was puzzled to know the origin of this pus.

After cleansing the vagina and uterus of pus, introduced a cloth tent with iodine and carbolic acid. Returned on Saturday; now no discharge, treatment repeated. Did not return for several weeks. When next examined with speculum and os opened with forceps, pus again gushed out as at first; same treatment. The following Saturday; no discharge in the vagina, but on opening the os pus gushed out again. Says she feels better; looks better and does not drag herself along as heretofore. There is evident want of contractility of the uterus to expell the pus, uterus is retroverted. Asks the opinion of the Academy upon the origin of the pus, as he has not encountered a similar case, and is at a loss to account for the copious discharge of pus.

Dr. Baird asked if there was any history of abcess?

Dr. T. replied he could find nothing to lead to belief of any inflammatory mischief. At last examination tried to pass sound into fallopian tube and used some force; there was some bleeding; uterine cavity is abnormally large. The case is peculiar in as much as there is no evidence of inflammatory or ulcerative condition of uterine cavity, so far as can be determined by usual means of exploration.

October 6th, 1873.

Dr. J. P. Logan, President, in the chair.

The President called attention of the Academy to the successful use of Wine of Pepsin with Bismuth in a case of *chronic cholera infantum* in child eighteen months old. There was extreme emaciation, and he thought the case almost hopeless. Gave a teaspoonful of the wine and five grains Bismuth, in a portion of food every four hours. There was a remarkable improvement in one week. Never saw such decided results from any other remedy in this class of cases.

Dr. J. G. Westmoreland inquired as regards the efficacy of Wine of Pepsin. Had been led to believe that it was not to be relied upon.

Dr. Logan had been prejudiced against the wine, and preferred the Pepsin powder in water. In this case, however, it acted well. The strength of the preparation is a grain of Pepsin to the teaspoonful of wine.

Dr. J. G. Westmoreland called attention to the fact that during the summer, and up to the present time, he has encountered some *peculiar cases of fever*. They are peculiar in their duration; being very protracted, but with no violent symptoms. The first case he would mention, without any dangerous symptom, went on for five weeks; and he has seen others drag along from four to six weeks, without any symptom to account for the protraction. The symptoms are less grave than he has encountered in any case of typhoid fever. Drs. Rauschenberg and Connally had met with cases running on for four or five weeks, with no decided intermission from the exhibition of quinine.

Dr. Westmoreland: Cases began intermittent, sometimes six hours intermission; prolonged by full doses quinine, and afterwards became continuous.

Dr. Logan: Considers them cases of typhoid fever; saw similar cases some years ago. Is of opinion there may be cases of typhoid fever without violent symptoms and with no local manifestations.

Dr. Westmoreland: Has seen cases of typhoid fever years ago, before and during the war, beginning as intermittent, though they continued not longer than twenty-one days; never saw a case of typhoid protracted so long as these cases are.

Dr. Rauschenberg: Has seen cases this summer such as described. They were broken up by quinine, for a few days and would then relapse; classed them with enteric fevers. They recover in four to five weeks. There was debility, no appetite. The temperature, noted in one case, was 41 deg. C. (106 deg. F.) coming down to natural temperature in the intermission.

Dr. Logan: Has seen only one case of fever during the summer, which he thought non-malarial, and that was the most distinctly marked case of typhoid fever he ever saw.

---

OCTOBER 20TH, 1873.

Dr. J. P. Logan, President, in the chair.

Dr. Taliaferro read an essay upon *Uterine Displacements.* He spoke first, of the supports of the uterus and its normal position in the pelvis. The uterus is poised upon the upper extremity of the vagina, between the bladder and rectum; its normal position being one of slight anteversion. The axis of its body corresponds with that of the superior strait, which is a line running from the umbilicus, or a little above it, to the coccyx. The upper surface of its larger end is generally on a level with the plane of the pelvic inlet, never higher, often lower, distant about three-fourths inch from the upper portion of the sacrum. The end of the cervix marks nearly the center of the pelvic cavity.

The cervix is maintained in position by the utero-illiac, utero-vesical, and utero-sacral processes; the body of the uterus by the broad ligaments, and round ligaments. The entire organ rests upon the upper extremity of the vagina which forms its main pillar of support. The uterus is also surrounded by areolar connective tissue, connecting it directly with the bladder and rectum. The vagina is closely united to the rectum by connective tissue, and abuts upon the perineum. The levator-ani muscle, with its pubo-coccygeal attachments, runs along the sides of the vagina and rectum, and supports the lower part of the vagina. This muscle, together with the obturator and pyriformis and the perineal body form the floor of the pelvis, which antagonizes the force exerted by the diaphragm.

The abdominal walls are also given by Dr. Matthews Duncan as a support, which, when they have not lost their tone, tend to prevent the pressure of the abdominal viscera upon the pelvic viscera. These supports are very elastic, and in its normal condition the uterus possesses great freedom of movement. There may be a physiological displacement of the uterus, caused by straining at defication, distended bladder, loaded rectum, coughing, sneezing, jumping, etc., which is not permanent, and the uterus returns to its normal position as soon as the displacing cause is removed. We must look for permanent displacements outside of accidents from the conditions mentioned.

As to the causes of uterine displacements Drs. Bennett and Thomas mention as one of the causes influences increasing the weight of the uterus, caused by congestions and inflammation. We have also, influences weakening uterine supports, viz: ruptured perineum, relaxation of vaginal walls by inflammation, etc.; wearing of tight clothing, which weakens the floor of the pelvis and likewise the abdominal walls, and relaxed and flaccid state of the submucous connective tissue.

We have also, influences pressing the uterus out of place, e.g., tight clothing, and especially tight corsets. Dr. Thomas, adds also, muscular efforts, lifting, etc., and repletion of the bladder. Dr. Taliaferro disagrees with him in this, as he considers these physiological conditions, which, although displacing the uterus temporarily, are not abnormal *per se.*

In treatment, Dr. T. considers displacements of the unimpregnated uterus as complications and effects of inflammations, and in order to effect a cure these causing conditions must first be removed. Inflammations, congestions, sub-involution, etc., must receive appropriate treatment; any abnormal condition of the vagina must be attended to; corsets and tight clothing must be removed; exercise, running, jumping, lifting, etc., are to be recommended.

Dr. W. F. Westmoreland asked what that force is which, according to the opinion of Dr. Matthews Duncan, the abdominal walls exert in support of the uterus and in preventing displacements.

Dr. J. G. Westmoreland believes the abdominal wall to be essential to the proper support of the pelvic viscera and their retention in proper position, and that whatever may be done to give tone and strength to the abdominal walls will tend to retain the pelvic viscera in position, prevent prolapsus uteri, etc.

Dr. W. F. Westmoreland could not see how the abdominal walls can retain the pelvic viscera in position. In many flaccid abdomens there are to be found uterine displacements, whilst in many others there are not, cannot see how flaccidity of the walls can have any effect in producing these displacements.

Dr. Taliaferro: When you have flaccid abdominal walls, other things being equal, you most frequently meet with prolapsus or other displacements of the uterus. It is the opinion of Dr. Matthews Duncan that the force within the abdominal walls serves to maintain the uterine organs *in situ*, through the agency of direct support to the abdominal viscera in general.

Dr. W. F. Westmoreland has often questioned—as female diseases are becoming very frequent now-a-days—whether or not the corset so frequently causes uterine troubles as is generally believed. Has been recently impressed with the belief that it is not so often the cause as is supposed. Frequently when advising women against the use of the corset; has been shown by them, that the corset is not at all tight; that the hand may be passed very easily up beneath it, and that the weight of the clothing is not on the waist but is supported by the hips. He would like to know if there would be any worse result from this than if the clothing were supported from the waist.

Dr. Taliaferro believes every woman likes to have a small waist, but they will not acknowledge that they wear tight corsets. You will find that many have worn corsets from girlhood, and in some cases the cartelages of the false ribs are over-lapped, whilst, in the normal position they are six to eight inches apart; at the same time they will not admit that their corsets have ever been worn tight. The French or "glove-fitting" corset fits to perfection. The steel and whalebone ribs act as splints, and so confine the muscles of the back and chest, that these become atrophied by pressure and absence of exercise. No woman will wear a corset unless sufficiently tight to give some sense of support to the weakened muscles, as they believe it is really of advantage to them. They are advised not to lift or do other kind of work which would bring their muscles into exercise, and for the want of it muscular tone is lost. Believes great injury is done by wearing corsets in girlhood, and that physicians are blamable if they do not advise mothers of the harm which may result.

Dr. W. F. Westmoreland: Does not deny that harm is done if the cartelages are over-lapped; but if a loose corset be worn supported upon the hips, is it not a support, and does it not rather prevent than cause a depression of the abdominal viscera? After interrogating a number of patients upon this point, he believes this to be true.

Dr. Taliaferro: They usually wear them tight enough to be a support. Admitting, however, that they are worn loose, so that when standing you may easily pass your hand beneath them, but when sitting the splints of steel and whalebone are bent inward upon the stomach, and pressing upon the abdominal viscera, push the latter downward into the pelvis. He does not admit that women wear their corsets loose. The muscles become so atrophied after a while that they cannot wear them loosely with comfort. He directs his patients to remove the corset entirely, and let the clothing be suspended from the shoulders, which are the natural supports, and that they bring the muscles into exercise by sweeping, lifting, etc.

